# YAP1 inhibits the senescence of alveolar epithelial cells by targeting Prdx3 to alleviate pulmonary fibrosis

**DOI:** 10.1038/s12276-024-01277-0

**Published:** 2024-07-01

**Authors:** Wei Su, Yingying Guo, Qianqian Wang, Lu Ma, Qing Zhang, Yuhan Zhang, Yiding Geng, Tongzhu Jin, Jiayu Guo, Ruoxuan Yang, Zhihui Niu, Lingxue Ren, Yan Wang, Zhiwei Ning, Wenyue Li, Wenxin He, Jian Sun, Tianyu Li, Zhixin Li, Hongli Shan, Haihai Liang

**Affiliations:** 1 Department of Pharmacology (State-Province Key Laboratories of Biomedicine-Pharmaceutics of China, Key Laboratory of Cardiovascular Research, Ministry of Education), College of Pharmacy, Harbin Medical University, Harbin, 150081 China; 2 Northern Translational Medicine Research and Cooperation Center, Heilongjiang Academy of Medical Sciences, Harbin Medical University, Harbin, 150081 China; 3 Department of Systems Biology, College of Bioinformatics Science and Technology, Harbin Medical University, Harbin, 150081 China; 4 Department of Thoracic Surgery, Shanghai Pulmonary Hospital, Tongji University, Shanghai, 200433 China; 5 Zhuhai People’s Hospital, Guangdong Provincial Key Laboratory of Tumor Interventional Diagnosis and Treatment, Zhuhai Hospital Affiliated with Jinan University, Jinan University, Zhuhai, 519000 Guangdong China; 6 Shanghai Frontiers Science Research Center for Druggability of Cardiovascular Noncoding RNA, Institute for Frontier Medical Technology, Shanghai University of Engineering Science, Shanghai, 201620 China; 7 Research Unit of Noninfectious Chronic Diseases in Frigid Zone (2019RU070), Chinese Academy of Medical Sciences, Harbin, 150081 China

**Keywords:** Senescence, Cell signalling

## Abstract

The senescence of alveolar type II (AT2) cells impedes self-repair of the lung epithelium and contributes to lung injury in the setting of idiopathic pulmonary fibrosis (IPF). Yes-associated protein 1 (YAP1) is essential for cell growth and organ development; however, the role of YAP1 in AT2 cells during pulmonary fibrosis is still unclear. YAP1 expression was found to be downregulated in the AT2 cells of PF patients. Deletion of YAP1 in AT2 cells resulted in lung injury, exacerbated extracellular matrix (ECM) deposition, and worsened lung function. In contrast, overexpression of YAP1 in AT2 cells promoted alveolar regeneration, mitigated pulmonary fibrosis, and improved lung function. In addition, overexpression of YAP1 alleviated bleomycin (BLM) -induced senescence of alveolar epithelial cells both in vivo and in vitro. Moreover, YAP1 promoted the expression of peroxiredoxin 3 (Prdx3) by directly interacting with TEAD1. Forced expression of Prdx3 inhibited senescence and improved mitochondrial dysfunction in BLM-treated MLE-12 cells, whereas depletion of Prdx3 partially abrogated the protective effect of YAP1. Furthermore, overexpression of Prdx3 facilitated self-repair of the injured lung and reduced ECM deposition, while silencing Prdx3 attenuated the antifibrotic effect of YAP1. In conclusion, this study demonstrated that YAP1 alleviates lung injury and pulmonary fibrosis by regulating Prdx3 expression to improve mitochondrial dysfunction and block senescence in AT2 cells, revealing a potential novel therapeutic strategy for pulmonary fibrosis.

## Alveolar YAP1: Key to lung regeneration and fibrosis prevention

Idiopathic pulmonary fibrosis is still not fully understood, and effective treatments are scarce. This study investigates the role of a protein, YAP1, in lung fibrosis. Researchers used mice and human lung samples to study how YAP1 influences lung cell aging and lung structure. The findings showed that increasing YAP1 levels in alveolar type II cells reduced lung fibrosis by improving the function of mitochondria and decreasing cell aging. Specifically, YAP1 worked through a pathway involving a molecule, Prdx3. However, reducing YAP1 levels worsened lung fibrosis. The researchers suggest that enhancing YAP1 activity in lung cells could be a potential treatment for lung fibrosis. This method targets lung cell aging and promotes healthy lung tissue repair. The results pave the way for developing treatments for IPF and possibly other similar lung diseases. This summary was initially drafted using artificial intelligence, then revised and fact-checked by the author.

## Introduction

Idiopathic pulmonary fibrosis (IPF) is a chronic disease characterized by scar formation in the lung parenchyma that causes loss of respiratory function and a consequent decrease in longevity^1,2^. Efforts are still being made to better understand the pathogenesis of IPF in the search for treatments for pulmonary fibrosis. Lung alveolar epithelial injury is a leading cause of multiple pulmonary diseases, including pulmonary fibrosis^3^. Under physiological conditions, alveolar type II (AT2) cells in injured lungs activate self-repair programs and promote their own proliferation and differentiation into alveolar type I (AT1) cells to maintain alveolar epithelial homeostasis^4^. However, persistent insults to the lungs lead to senescence of AT2 cells and maladaptive lung repair, ultimately resulting in pulmonary fibrosis^5^. It has been reported that the senolytic drug combination of dasatinib and quercetin inhibited senescence in the alveolar epithelium, thus attenuating experimental pulmonary fibrosis in vitro^6^. This indicates that blocking the senescence of AT2 cells might be a therapeutic strategy for pulmonary fibrosis.

Cellular senescence, defined as permanent arrest of the cell cycle, can be triggered by multiple stress stimuli^7^. Senescent cells exhibit an enlarged and flattened morphology and increased expression of β-galactosidase^8^. Although senescent cells remain in the stationary phase of the cell cycle, they still express various cytokines through the activation of a program known as the senescence-associated secretory phenotype (SASP)^9^. Mitochondrial dysfunction might be both a cause and a result of senescence and plays a critical role in inducing and sustaining the senescence phenotype^10^. The features of mitochondrial dysfunction, such as a reduced respiratory capacity, a decreased mitochondrial membrane potential, and overproduction of reactive oxygen species (ROS), contribute to cellular senescence. Recent research has revealed that melatonin treatment inhibits premature senescence in A549 cells by alleviating mitochondrial dysfunction^11^.

The Hippo signaling pathway was originally identified in research on tumor suppressor genes in *Drosophila*^12^. YAP1 is a vital downstream effector of Hippo signaling that plays a critical role in modulating organ size, tissue homeostasis, and regeneration^13^. Due to the lack of DNA-binding domains, YAP1 exerts its effects by interacting with DNA-binding proteins, among which TEAD1 is the most frequent interacting partner. It has been reported that the YAP1/TEAD1 complex mediates mitochondrial biogenesis and angiogenesis by directly targeting PGC1α^14^. Moreover, in osteoarthritis, YAP1 functions as a senescence inhibitor by upregulating the expression of FOXD1^15^. These findings indicate that YAP1 may inhibit cellular senescence by improving mitochondrial function.

In this study, YAP1 expression was found to be increased in the lung fibroblasts of patients with pulmonary fibrosis and in BLM-treated mice, which was in line with our previous study^16^. Moreover, we found downregulation of YAP1 in AT2 cells in the lungs of IPF patients. Overexpression of YAP1 prevented lung senescence, restored lung structure, and inhibited pulmonary fibrosis. More importantly, we revealed that YAP1/TEAD1 improved mitochondrial function by transcriptionally activating peroxiredoxin 3 (Prdx3), thereby suppressing the senescence of AT2 cells. Overall, we propose the role of the YAP1/TEAD1-Prdx3 axis in regulating AT2 cell senescence and pulmonary fibrosis, revealing a novel strategy for the treatment of pulmonary fibrosis.

## Materials and methods

### Animal models

All animal experiments and human samples were approved by the Harbin Medical University College of Pharmacy Ethical Committee (A-IRB3001721) and conformed to the Declaration of Helsinki. YAP1^fl/fl^ and Sftpc-CreERT2 mice were purchased from The Jackson Laboratory (Bar Harbor, USA), YAP1^S127A^ transgenic mice were obtained from Cyagen Biosciences (Guangzhou, China), and wild-type C57BL/6 mice (22–25 g) were purchased from Liaoning Changsheng Biotechnology Co., Ltd. (Benxi, China). YAP1^fl/fl^ and Sftpc-CreERT2 mice were crossbred into YAP1^fl/fl^; Sftpc-CreERT2 transgenic mice, hereafter referred to as YAP1-cKO mice. YAP1^S127A^ and Sftpc-CreERT2 mice were crossbred to produce YAP1^S127A^; Sftpc-CreERT2 transgenic mice, hereafter referred to as YAP1-cKI mice. The transgenic mice were treated with 75 mg/kg tamoxifen for 7 days and then allowed to rest for more than 7 days to avoid adverse effects.

To establish the pulmonary fibrosis models, 3 mg/kg bleomycin (BLM) was intratracheally administered to mice, and the lungs were harvested after 21 days.

### Western blot analysis

Proteins were extracted from cells of the lung lobes and then separated by electrophoresis on SDS‒PAGE gels. Next, the proteins were transferred onto nitrocellulose membranes (Pall Life Science, USA), which were blocked with 5% skim milk and incubated with primary antibodies at 4 °C overnight. An anti-β-actin antibody (Proteintech, 66009-1-Ig, Wuhan, China) was used as an internal control; antibodies were against YAP1 (Proteintech, 66900-1-Ig), Fn1 (Proteintech, 15613-1-AP, α-SMA (Abcam Inc., ab7817), p21 (Abcam Inc., ab188224), p16 (Wanlei, WL01418, Shenyang, China), Prdx3 (Proteintech, 10664-1-AP), Bax (Proteintech, 50599-2-Ig), Cyt-C (Proteintech, 12245-1-AP), Drp1 (Affinity, DF7037, Changzhou, China) and Mfn2 (Affinity, DF8106) were also used. The membranes were then incubated with the corresponding secondary antibodies, and protein band densities were quantified by Odyssey Image Studio Version 5.2.

### Quantitative real-time PCR

Total RNA was extracted using TRIzol reagent (Invitrogen, Carlsbad, USA), and a reverse transcription kit (TransGen Biotech, Beijing, China) was used to synthesize complementary DNA. qRT‒PCR was performed using an ABI 7500 FAST real-time PCR system according to the experimental protocol with SYBER GREEN (Roche, Basel, Switzerland). Relative expression was determined using the Ct value of the gene of interest normalized to the Ct value of GAPDH.

### Tissue immunofluorescence staining

Lung lobes were embedded in OCT (Sakura, Finetek, Japan) and sliced into 5 μm-thick sections. The frozen sections were fixed with acetone, after which peroxidase activity was blocked by treatment with 3% H_2_O_2_, and the tissue sections were then incubated with primary antibodies specific for Sftpc (Santa Cruz, sc-518029, USA) (Proteintech, 10774-1-AP), YAP1 (Proteintech, 66900-1-Ig), and Aqp5 (Affinity, AF5169). DAPI was used to label nuclei. Images were acquired using a confocal microscope (FluoView FV10i, Olympus, Tokyo, Japan).

### Pulmonary function test

Pulmonary function was assessed using the FlexiVent Pulmonary System (SCIREQ Scientific, Canada). Mice were anesthetized with pentobarbital sodium, and a tracheal cannula was then inserted. Parameters reflecting lung function were assessed.

### Micro-CT

Mice were scanned using a Hiscan XM Micro CT scanner (Suzhou Hiscan Information Technology Co., Ltd.). Regarding the scan conditions, the X-ray tube settings were 60 kV and 134 µA, and images were acquired at a resolution of 50 μm. A 0.5° rotation step through a 360° angular range with a 50 ms exposure time per step was used. The images were reconstructed using Hiscan Reconstruct software (version 3.0, Suzhou Hiscan Information Technology Co., Ltd.) and analyzed using Hiscan Analyzer software (version 3.0, Suzhou Hiscan Information Technology Co., Ltd.).

### Histopathological analysis

Lung tissues were dehydrated, embedded in paraffin, and sliced into 5 μm-thick sections. The paraffin-embedded sections were then dewaxed and rehydrated for subsequent experiments. Lung sections were stained using Masson staining or HE staining kits purchased from Solarbio Life Science (Beijing, China). For immunohistochemistry, lung sections were soaked in 3% H_2_O_2_ solution to block endogenous peroxidase activity. After antigen retrieval and blocking, the lung slices were incubated with primary antibodies at 4 °C overnight. The next day, the lung tissues were incubated with secondary antibodies and stained with DAB (ZSGB-BIO, Beijing, China).

### Hydroxyproline content assay

The hydroxyproline content was measured using a kit purchased from Nanjing Jiancheng Bioengineering Institute (Nanjing, China) according to the manufacturer’s protocol.

### SA-β-galactosidase staining

SA-β-Galactosidase activity in lung sections and cells was measured using an SA-β-galactosidase staining kit (Beyotime Biotechnology, Shanghai, China). After fixation, lung tissues and cells were incubated with the dye mixture at 37 °C for 24 h. Images were obtained using a microscope (Olympus, Tokyo, Japan).

### iTRAQ proteomic analysis

MLE-12 cells were cultured in 10 cm Petri dishes and transfected with a vector or YAP1 overexpression plasmid. The cells were collected when they had proliferated to form a single layer. iTRAQ proteomic analysis was performed by Wuhan Genecreate Biological Engineering Co., Ltd. (Wuhan, China).

### ChIP sequencing

MLE-12 cells were cultured in 10 cm Petri dishes and transfected with the TEAD1 overexpression plasmid. When the cells had proliferated to 90% confluence, the medium was removed, fresh medium was added, and the cells were cultured for 30 min. An appropriate formaldehyde solution was added for 10 min, and glycine was added to terminate the crosslinking reaction. The cells were then collected, and ChIP sequencing was performed at Wuhan SeqHealth Tech Co., Ltd. (Wuhan, China).

### ChIP assay

ChIP assays were performed using an anti-TEAD1 antibody (Abcam Inc., USA). Ten percent of the chromatin not subjected to immunoprecipitation was used as input for the positive control, and reactions with a nonspecific antibody (rabbit IgG; BD Biosciences) served as negative controls.

### Luciferase assay

The pGL3-Prdx3-promoter vector was divided into five different segments, which were obtained by inserting the promoter region of Prdx3 (− 2000 to + 500 bp) into the pGL3-basic vector, and the resulting plasmids used for transfection were pGL3-Prdx3-1, pGL3-Prdx3-2, pGL3-Prdx3-3, pGL3-Prdx3-4, pGL3-Prdx3-5, and pGL3-basic. MLE-12 cells were cotransfected with the vector or YAP1 plasmid and pGL3-Prdx3-1, pGL3-Prdx3-2, pGL3-Prdx3-3, pGL3-Prdx3-4, pGL3-Prdx3-5, or pGL3-basic. The cells were lysed using passive lysis buffer, and the lysates were analyzed using the Dual-Luciferase Reporter Assay System (Promega, GloMax, Sunnyvale, USA).

### Intracellular ROS detection

The intracellular ROS content was tested using a reactive oxygen species assay kit obtained from Beyotime Biotechnology (Shanghai, China). Cells were incubated first with the DCFH-DA probe at 37 °C in a cell incubator for 30 min and then with Hoechst 33342 (Solarbio Life Science, Beijing, China) for 10 min. Images were acquired using an inverted microscope (Olympus, Tokyo, Japan).

### Mitochondrial ROS measurement

The mitochondrial ROS content was measured using a kit purchased from BestBio (Nanjing, China). Cells were incubated first with the preheated probe solution at 37 °C in a cell incubator for 30 min and then with Hoechst 33342 for 10 min. Images were acquired using an inverted microscope (Olympus, Tokyo, Japan).

### Mitochondrial membrane potential test

Cells were incubated first with tetramethylrhodamine (TMRM) at 37 °C in a cell incubator for 30 min and then with Hoechst 33342 for 10 min. Images were acquired using an inverted microscope (Olympus, Tokyo, Japan).

### Mito tracker analysis

Cells were incubated first with the MitoTracker^®^ Red FM probe (Invitrogen, Carlsbad, CA, USA) at 37 °C in a cell incubator for 30 min and then with Hoechst 33342 for 10 min. Images were acquired using a confocal microscope (FluoView FV10i, Olympus, Japan).

### Cell culture and transfection

MLE-12 cells were cultured in DMEM supplemented with 10% FBS and 1% penicillin‒streptomycin. During the transfection process, the complete medium was replaced with DMEM. Next, the plasmid or siRNA was mixed with Lipofectamine 2000 (Life Technologies, USA) and Opti-MEM (Invitrogen, USA), added to 6-well plates, and coincubated for 6 h.

### Statistical analysis

The data are presented as the means ± SEMs. One-way analysis of variance (ANOVA) followed by the Bonferroni test or Dunnett’s post hoc test was used for comparisons among more than two groups. A two-tailed value of *P* < 0.05 was considered to indicate statistical significance. The data were analyzed using GraphPad Prism 8.0.

## Results

### Downregulated expression of YAP1 in IPF

**Fig. 1:**
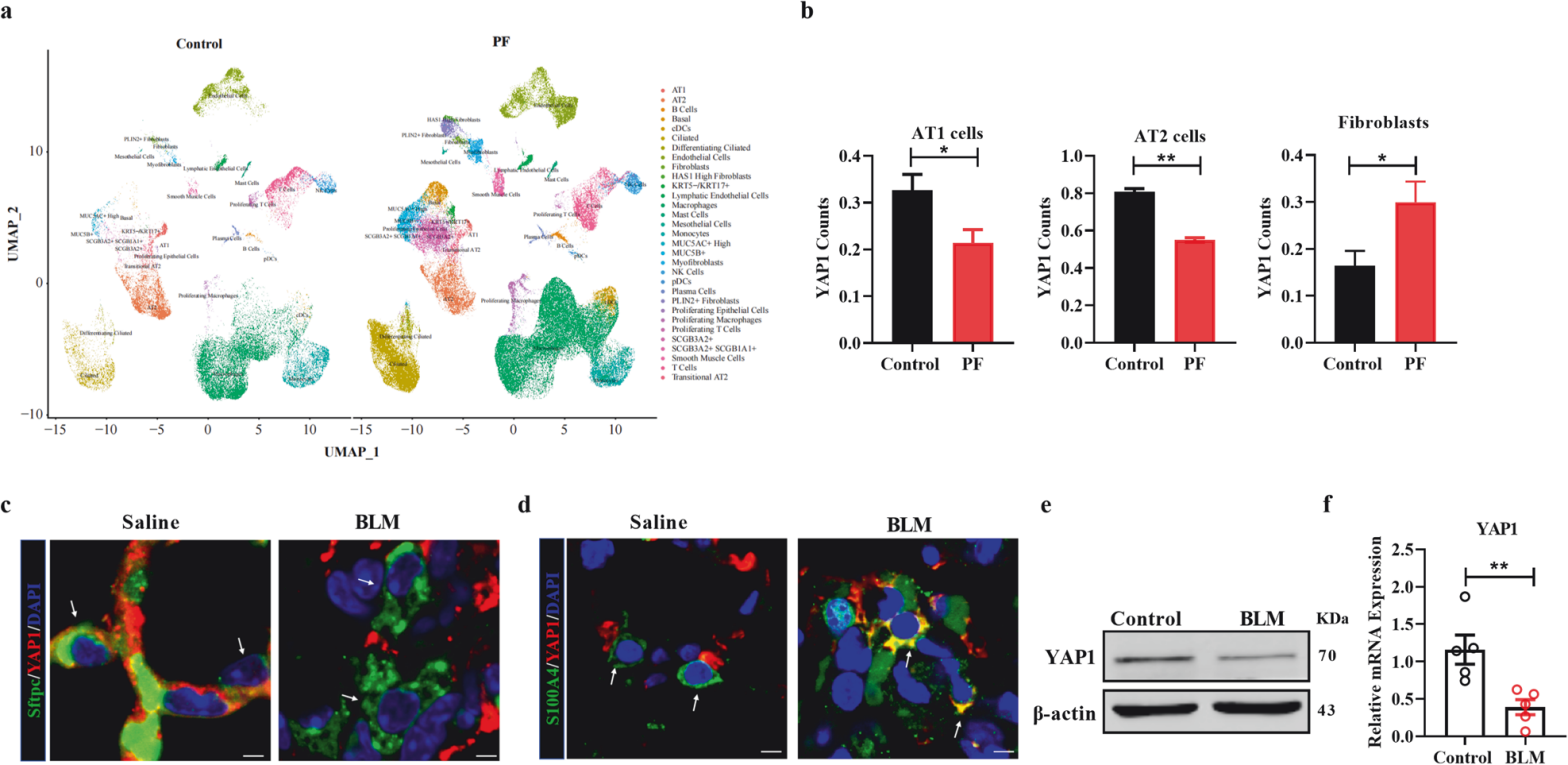
YAP1 expression is decreased in AT2 cells during pulmonary fibrosis. **a**, **b** Bioinformatics analysis of the expression of YAP1 in AT1 cells, AT2 cells and fibroblasts from healthy donors and IPF patients (GSE135893). **c**, **d** Representative images showing YAP1 (red), Sftpc (green), S100A4 (green) and nuclei (blue) in lung sections from mice treated with saline or BLM. Scale bars, 10 μm. **e** Western blot analysis of YAP1 expression in MLE-12 cells after BLM induction; *n* = 6. **f** qRT‒PCR analysis of YAP1 mRNA expression in MLE-12 cells treated with BLM; *n* = 5. The data are presented as the means ± SEMs. **P < 0.01.

First, we aimed to explore the expression of YAP1 during pulmonary fibrosis. A set of single-cell data from IPF patients (GSE135893) was obtained, and analysis of this dataset revealed significantly decreased expression of YAP1 in AT1 and AT2 cells from IPF patients compared with those from control donors, while the expression of YAP1 was significantly increased in lung fibroblasts, in accordance with our previous finding that the upregulation of YAP1 in lung fibroblasts promoted pulmonary fibrosis (Fig. 1a, b). As the loss and senescence of AT2 cells are leading causes of pulmonary fibrosis and the poor proliferation of AT1 cells^17^, we focused on the role of YAP1 in AT2 cells during fibrosis.

We established a mouse model of pulmonary fibrosis by intratracheal BLM administration to further explore the function of YAP1. To validate the expression of YAP1 in AT2 cells, we performed dual immunofluorescence staining on frozen lung sections, which revealed decreased colocalization of YAP1 and Sftpc in BLM-treated mice (Fig. 1c) but increased expression of YAP1 in S100A4-labeled lung fibroblasts (Fig. 1d). Additionally, western blot and qRT‒PCR analyses demonstrated a sharp reduction in YAP1 expression in BLM-treated MLE-12 (mouse AT2) cells (Fig. 1e, f). Collectively, these results indicated that the expression of YAP1 in AT2 cells is decreased during pulmonary fibrosis, which may contribute to the progression of IPF.

### Deficiency of YAP1 in AT2 cells exacerbates pulmonary fibrosis

**Fig. 2:**
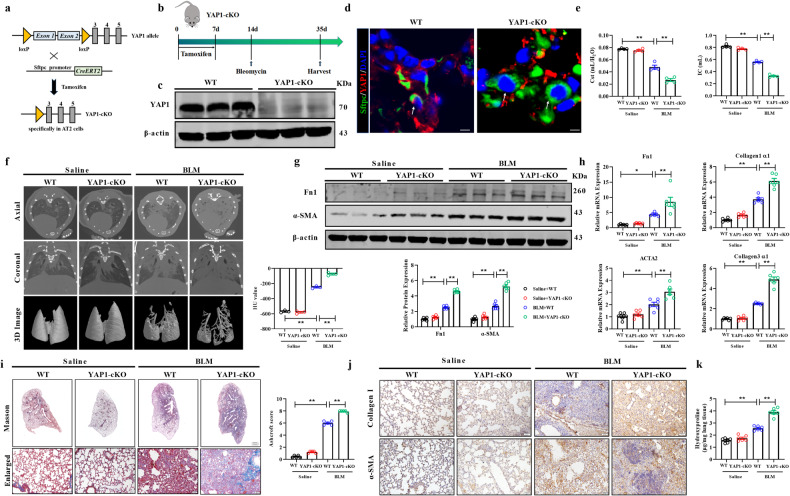
YAP1 knockout in AT2 cells exacerbates BLM-induced pulmonary fibrosis. **a** Method used to generate YAP1-cKO mice. YAP1^fl/fl^ mice were hybridized with Sftpc-CreERT2 mice. **b** Schematic diagram demonstrating the deletion of YAP1 in BLM-induced mouse pulmonary fibrosis models. **c** Western blot analysis of YAP1 expression in the lungs of YAP1-cKO mice; *n* = 6. **d** Representative images showing YAP1 (red), Sftpc (green), and nuclei (blue) in lung sections from WT and YAP1-cKO mice. Scale bars, 10 μm. **e** Pulmonary function parameters such as Cst (static compliance) and IC (inspiratory capacity) in the different groups, n = 4. **f** Micro-CT images showing the axial plane (upper panels) and corresponding coronal plane (middle panels) in the various groups. The bottom panels show images of three-dimensional reconstructions of lung tissues based on the density, *n* = 3. **g** Western blot analysis of the expression of Fn1 and α-SMA in lung homogenates from BLM-induced WT and YAP1-cKO mice; *n* = 6. **h** qRT‒PCR analysis of the mRNA expression of Fn1, Collagen 1α1, Collagen 3α1 and ACTA2 in WT and YAP1-cKO mice treated with saline or BLM; *n* = 6. **i**, **j** Masson’s trichrome staining and immunohistochemical staining of paraffin-embedded lung sections from WT or YAP1-cKO mice treated with saline or BLM; *n* = 5. Scale bars, 50 μm. **k** Hydroxyproline content in WT and YAP1-cKO mice treated with saline or BLM; *n* = 6. The data are presented as the means ± SEMs. *P < 0.05, ** *P* < 0.01.

We then established transgenic mice with conditional YAP1 knockout in AT2 cells by crossing YAP1^fl/fl^ mice with Sftpc-labeled Cre (Sftpc-Cre) mice to explore the role of YAP1 in pulmonary fibrosis (Fig. 2a, b). The knockout efficiency of YAP1 was validated using western blot and immunofluorescence analyses (Fig. 2c, d). The results indicated that YAP1 expression was significantly lower in YAP1-cKO mice than in WT mice, which confirmed the successful knockout of YAP1 in AT2 cells. The pulmonary function test revealed marked reductions in lung compliance and IC in wild-type (WT) mice treated with BLM, and YAP1-cKO mice exhibited an even lower respiratory capacity after BLM administration (Fig. 2e). Micro-CT analysis also showed that YAP1 knockdown in AT2 cells exacerbated pulmonary fibrosis after BLM induction (Fig. 2f). Western blot and qRT‒PCR analyses revealed significantly increased proteins and mRNA expression of fibrosis-associated factors, such as Fn1 and α-SMA, in WT mice treated with BLM. Compared with the counterpart WT mice, BLM-treated YAP1-cKO mice exhibited increased expression levels of fibrotic markers such as Fn1 and α-SMA (Fig. 2g, h). Obvious increases in collagen deposition and the degree of pulmonary fibrosis after BLM challenge were detected in YAP1-cKO mice compared to WT mice by Masson’s trichrome staining, immunohistochemical analysis, and hydroxyproline content measurement (Fig. 2i, k). In line with the above results, our findings showed that knockout of YAP1 in AT2 cells exacerbated pulmonary fibrosis after BLM challenge.

### Overexpression of YAP1 in AT2 cells inhibits pulmonary fibrosis after BLM induction

**Fig. 3:**
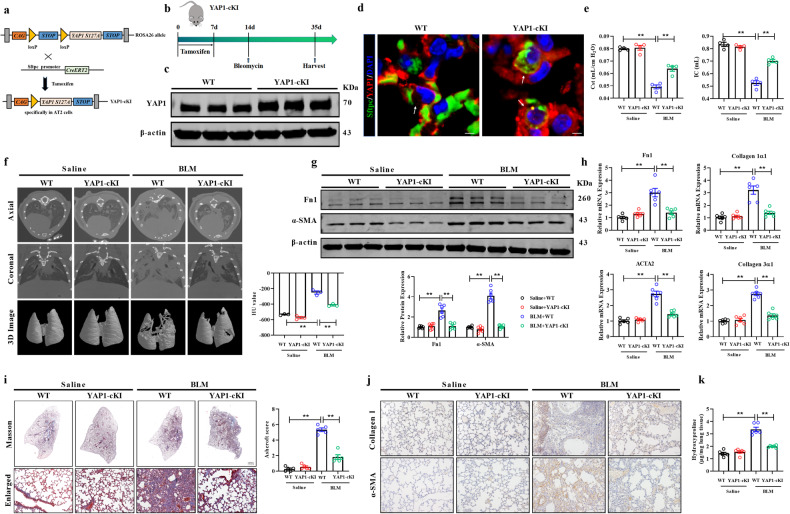
Overexpression of YAP1 in AT2 cells mitigated pulmonary fibrosis. **a** Breeding method for generating YAP1-cKI mice. YAP1^S127A^ mice were hybridized with Sftpc-CreERT2 mice. **b** Schematic diagram showing the overexpression of YAP1 in the BLM-induced mouse model of pulmonary fibrosis. **c** Western blot analysis of the expression of YAP1 in AT2 cells isolated from YAP1-cKI mice; *n* = 6. **d** Representative images showing YAP1 (red), Sftpc (green), and nuclei (blue) in lung sections from WT and YAP1-cKI mice. Scale bars, 10 μm. **e** Pulmonary function parameters, including the Cst and IC, were measured in the different groups; *n* = 4. **f** Micro-CT images of the axial plane (upper panels) and corresponding coronal plane (middle panels) in separate groups are shown. The bottom panels show images of three-dimensional reconstructions of lung tissues based on the density; *n* = 3. **g** The expression of Fn1 and α-SMA in lung homogenates from BLM-induced WT and YAP1-cKI mice was assessed by western blot analysis; *n* = 6. **h** qRT‒PCR analysis of the mRNA expression of Fn1, Collagen1α1, Collagen 3α1 and ACTA2 in WT and YAP1-cKI mice treated with saline or BLM; *n* = 6. **i**, **j** Masson’s trichrome staining and immunohistochemical staining of paraffin lung sections from WT or YAP1-cKI mice administered saline or BLM; *n* = 5. Scale bars, 50 μm. **k** Hydroxyproline content in WT and YAP1-cKI mice treated with saline or BLM; *n* = 6. The data are presented as the means ± SEMs. ** *P* < 0.01.

Then, we generated mice with conditional overexpression of YAP1 in AT2 cells (YAP1-cKI mice) by crossing YAP1S127A (a vector containing a YAP1 DNA sequence without a phosphorylation site via the S127A mutation) mice with Sftpc-Cre mice to explore whether the forced expression of YAP1 in AT2 cells can inhibit experimental pulmonary fibrosis (Fig. 3a, b). Western blot and immunofluorescence analyses were performed to verify the overexpression of YAP1 in AT2 cells (Fig. 3c, d). Pulmonary function tests and micro-CT analysis indicated that forced expression of YAP1 in AT2 cells significantly restored lung compliance and attenuated lung structural remodeling (Fig. 3e, f). Moreover, we found that the overexpression of YAP1 in AT2 cells markedly suppressed the expression of fibrosis-related proteins and genes such as Fn1 and α-SMA following BLM treatment, as seen in YAP1-cKI mice compared to WT mice (Fig. 3g, h). Furthermore, overexpression of YAP1 in AT2 cells significantly inhibited the deposition of collagen, suppressed the activation of myofibroblasts, and decreased the content of hydroxyproline (Fig. 3i, k). Taken together, these findings demonstrated that overexpression of YAP1 in AT2 cells inhibited BLM-induced pulmonary fibrosis.

### Role of YAP1 in BLM-induced lung injury and senescence

**Fig. 4:**
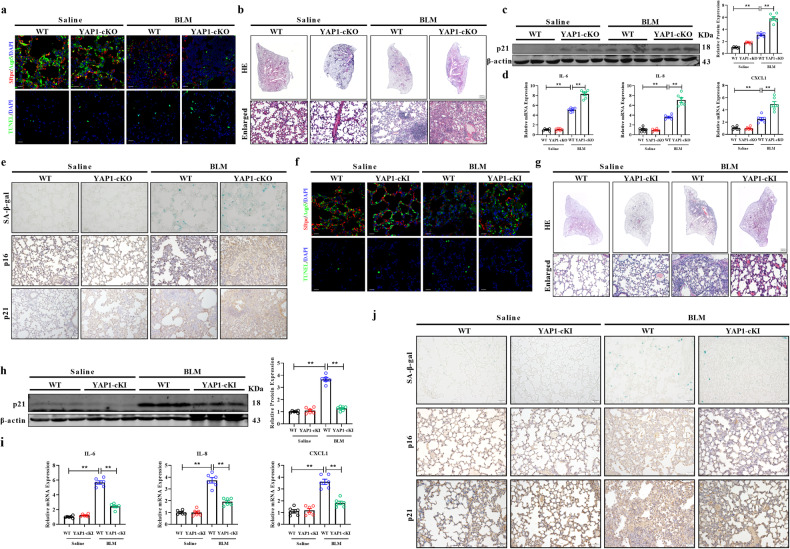
Function of YAP1 in BLM-induced lung injury and senescence. **a** Representative images showing Sftpc (red), Aqp5 (green), and nuclei (blue) in frozen lung sections from WT and YAP1-cKO mice. TUNEL assay of apoptosis, with TUNEL-positive cells indicated by green fluorescence and nuclei marked with DAPI. Scale bars, 20 μm. **b** Images of HE staining in tissues from WT and YAP1-cKO mice treated with saline or BLM. Scale bars, 50 μm. **c** Western blot analysis of p21 protein expression in WT and YAP1-cKO mice treated with saline or BLM; *n* = 6. **d** qRT‒PCR analysis of the mRNA expression of IL-6, IL-8, and CXCL1; *n* = 6. **e** SA-β-gal and IHC staining in lung sections from WT and YAP1-cKO mice treated with saline or BLM. Scale bars, 50 μm. **f** Representative images of frozen lung sections from WT and YAP1-cKI mice, with fluorescence indicating the following: Sftpc (red), Aqp5 (green), and nuclei (blue). TUNEL-positive cells are indicated by green fluorescence, and nuclei are indicated by blue fluorescence. Scale bars, 20 μm. **g** Images of HE staining in tissues from WT and YAP1-cKI mice treated with saline or BLM. Scale bars, 50 μm. **h** Western blot analysis of p21 protein expression in WT and YAP1-cKI mice treated with saline or BLM; *n* = 6. **i** qRT‒PCR analysis of the mRNA expression of IL-6, IL-8, and CXCL1; *n* = 6. **j** SA-β-gal and IHC staining in lung sections from WT and YAP1-cKI mice treated with saline or BLM. Scale bars, 50 μm. The data are presented as the means ± SEMs. **P < 0.01.

It is well known that the senescence and apoptosis of AT2 cells disrupt lung homeostasis, ultimately leading to pulmonary fibrosis^18^. Thus, we hypothesized that YAP1 prevents pulmonary fibrosis by inhibiting the senescence of AT2 cells and promoting the regeneration of pulmonary alveoli. As shown in Fig. 4a, compared to that in WT mice, the number of Aqp5/Sftpc-labeled AT1/AT2 cells decreased markedly in YAP1-cKO mice after BLM administration. Moreover, TUNEL assays showed that knockout of YAP1 in AT2 cells promoted apoptosis (Fig. 4a). Moreover, HE staining revealed exacerbated damage to the lung structure and inflammatory infiltration in YAP1-cKO mice treated with BLM (Fig. 4b). Western blotting revealed greater p21 expression in YAP1-cKO mice than in WT mice after BLM treatment (Fig. 4c). Accordingly, YAP1-cKO mice treated with BLM exhibited dramatically elevated expression of SASP factors, such as IL-6, IL-8, and CXCL1 (Fig. 4d). IHC staining revealed increased expression levels of senescence-associated proteins, including p16 and p21, in YAP1-cKO mice treated with BLM; similarly, senescence-associated β-galactosidase activity was greater in YAP1-cKO mice than in WT mice treated with BLM (Fig. 4e).

In contrast, the overexpression of YAP1 in AT2 cells facilitated the regeneration of lung alveoli and inhibited AT2 cell apoptosis (Fig. 4f). Moreover, compared to the counterpart WT mice, BLM-treated YAP1-cKI mice exhibited attenuation of lung remodeling and inflammatory responses (Fig. 4g). Moreover, decreased p21 and SASP factor expression was detected in BLM-treated YAP1-cKI mice by western blotting and qRT‒PCR (Fig. 4h, i). IHC staining and SA-β-gal assays also suggested a decrease in cellular senescence in YAP1-cKI mice following BLM induction (Fig. 4j). Taken together, these results indicate that YAP1 deficiency in AT2 cells promotes cellular senescence and apoptosis in the lungs, whereas YAP1 overexpression in AT2 cells inhibits senescence and facilitates lung regeneration after BLM injury.

### YAP1 transcriptionally activates Prdx3 by interacting with TEAD1

**Fig. 5:**
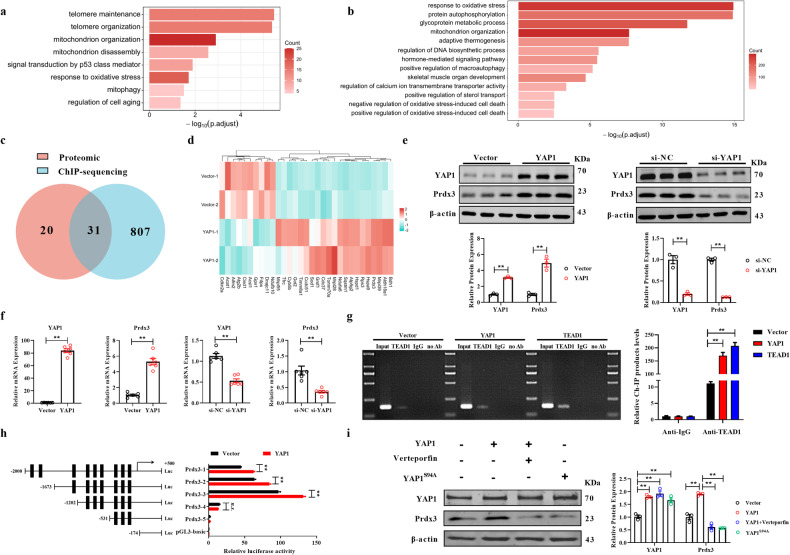
YAP1/TEAD1 directly targets Prdx3. **a** Proteomic analysis showing that signaling pathways were modulated by YAP1. **b** ChIP sequencing analysis results demonstrating the signaling pathways regulated by TEAD1. **c**, **d** Heatmap showing the expression of overlapping significantly altered genes identified by integrating the proteomics and ChIP sequencing data. **e**, **f** Western blot and qRT‒PCR analyses of the expression of Prdx3 in MLE-12 cells transfected with the YAP1 plasmid or si-YAP1, *n* = 3. **g** ChIP assay showing the relative ChIP product abundances in MLE-12 cells transfected with the YAP1 or TEAD1 plasmid; *n* = 3. **h** Relative luciferase activity was measured in MLE-12 cells transfected with various fragments of the Prdx3 promoter; *n* = 3. **i** Western blot showing the expression levels of Prdx3 in YAP1-overexpressing MLE-12 cells treated with verteporfin or expressing YAP1^S94A^; *n* = 3. The data are presented as the means ± SEMs. ** *P* < 0.01.

As a transcriptional coactivator, YAP1 may perform its function by entering the nucleus and binding to the transcription factor TEAD1 to regulate the expression of downstream genes^19^. Therefore, we further explored the underlying mechanism by which YAP1 inhibits senescence. ChIP-seq and proteomic analyses showed that YAP1 modulated mitochondrial organization and the response to oxidative stress-related signaling **(**Fig. 5a, b). Then, we integrated the ChIP-seq and proteomics data, and 31 overlapping genes were found. Then, we found that peroxiredoxin-3 (Prdx3), a mitochondrial antioxidant, exhibited a strong regulatory relationship with YAP1 (Fig. 5c, d). Western blot and qRT‒PCR analyses demonstrated that overexpression of YAP1 increased the protein and mRNA expression of Prdx3, whereas silencing of YAP1 exerted the opposite effects (Fig. 5e, f). A ChIP assay was used to validate the regulatory effect of YAP1/TEAD1 on the Prdx3 promoter, and the results showed that the presence of YAP1/TEAD1 significantly increased the relative abundance of ChIP products, substantiating the modulatory relationship of the YAP1/TEAD1-Prdx3 axis (Fig. 5g). Furthermore, the binding site of YAP1/TEAD1 in the Prdx3 promoter was determined using a luciferase assay: 9 binding sites in the Prdx3 promoter were predicted by JASPAR, and the Prdx3 promoter was subsequently divided into 4 parts. YAP1/TEAD1 had a much weaker regulatory effect on Prdx3-4 than on Prdx3-1, Prdx3-2, or Prdx3-3, which means that the sites at which YAP1/TEAD binds to the promoter of Prdx3 are in the region between positions -1202 and -531 (Fig. 5h). More importantly, blockade of the YAP1/TEAD1 interaction by verteporfin treatment or the YAP1^S94A^ mutation decreased the ability of YAP1 to upregulate Prdx3 (Fig. 5i).

### Prdx3 is necessary for the protective effect of YAP1 against senescence and mitochondrial dysfunction

Since mitochondrial dysfunction is a precipitating factor for cellular senescence, we explored the effect of Prdx3 on mitochondrial function and senescence in MLE-12 cells after BLM induction. As shown in Supplementary Fig. 1a, b, Prdx3 significantly inhibited BLM-induced cellular senescence by downregulating the expression of p16 and p21 and decreasing the activity of SA-β-gal. Moreover, Prdx3 overexpression markedly suppressed MLE-12 cell apoptosis (Supplementary Fig. 1c). The formation of γH_2_AX foci, a hallmark of DNA damage and senescence, was also inhibited in MLE-12 cells overexpressing Prdx3 after BLM induction (Supplementary Fig. 1d). BLM treatment dramatically increased the expression of Drp1 and inhibited the expression of Mfn2, whereas these effects were reversed by transfection of the Prdx3 overexpression vector (Supplementary Fig. 1e). ROS measurement demonstrated reduced levels of cytosolic and mitochondrial ROS in MLE-12 cells transfected with the Prdx3 overexpression vector, indicating that elevated expression of Prdx3 could reduce intracellular ROS accumulation (Supplementary Fig. 1f). Accordingly, overexpression of Prdx3 restored the mitochondrial membrane potential and maintained mitochondrial length and morphology (Supplementary Fig. 1g, h). Moreover, overexpression of Prdx3 increased oxygen consumption in BLM-treated MLE-12 cells (Supplementary Fig. 1i). Collectively, these findings suggest that Prdx3 plays a protective role by inhibiting BLM-induced cellular senescence and mitochondrial dysfunction.

**Fig. 6:**
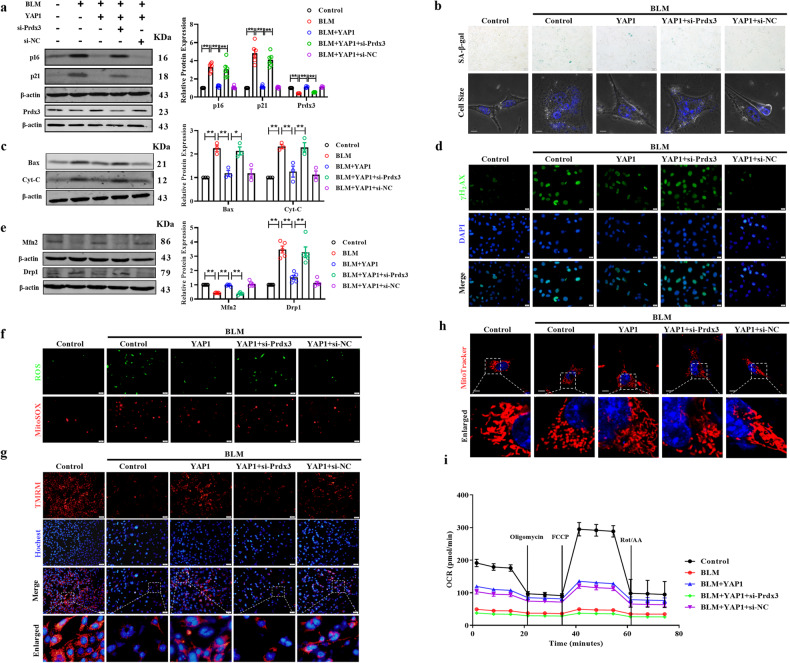
Silencing of Prdx3 abolishes the effects of YAP1 on senescence and mitochondrial dysfunction. **a** Western blot analysis of p16 and p21 expression; *n* = 6. **b** SA-β-gal staining to evaluate senescence in MLE-12 cells transfected with the YAP1 overexpression plasmid or si-Prdx3 after BLM treatment; *n* = 6. Scale bars, 50 μm. The cell size was determined by confocal microscopy, and nuclei were stained with Hoechst 33342 (blue). Scale bars, 10 μm. **c** Western blot results showing the protein expression of Bax and Cyt-C in MLE-12 cells transfected with the YAP1 overexpression plasmid or si-Prdx3 after BLM treatment; *n* = 3. **d** Immunofluorescence staining of γH_2_AX in MLE-12 cells with YAP1 overexpression or Prdx3 silencing following BLM induction. Scale bars, 20 μm. **e** The protein levels of Drp1 and Mfn2 were measured by western blotting, *n* = 5. **f** Cytosolic and mitochondrial ROS levels in Prdx3-overexpressing MLE-12 cells after BLM administration were measured using a DCFH-DA probe and MitoSOX. Scale bars, 50 μm. **g** Representative images of MLE-12 cells stained with TMRM (red) and DAPI (nuclei; blue). Scale bars, 10 μm. **h** Representative images acquired by confocal microscopy showing morphological changes in mitochondria. Scale bars, 10 μm. **i** The O_2_ consumption rate was assessed using a Seahorse mitochondrial stress test. The data are presented as the means ± SEMs. *P < 0.05, **P < 0.01.

Then, we explored whether YAP1 exerts its protective effect by regulating Prdx3. Western blotting showed that Prdx3 silencing partially abrogated the inhibitory effect of YAP1 on senescence and that overexpression of YAP1 significantly downregulated the expression of senescence-associated markers such as p16 and p21, an effect that was abolished by si-Prdx3 transfection (Fig. 6a). SA-β-gal activity was elevated in YAP1-overexpressing cells transfected with si-Prdx3; moreover, the decrease in the cell size caused by YAP1 overexpression was abolished after silencing of Prdx3 (Fig. 6b). In addition, knockdown of Prdx3 alleviated the inhibitory effect of YAP1 on the expression of Bax and Cyt-C, indicating that YAP1 modulates apoptosis through Prdx3 (Fig. 6c). Immunofluorescence assays revealed that YAP1 overexpression decreased the fluorescence intensity of γH_2_AX and that this decrease was abolished by si-Prdx3, suggesting the antisenescence function of the YAP1-Prdx3 axis (Fig. 6d and Supplementary Fig. 2a). Similarly, forced expression of YAP1 increased the expression of Mfn2 and downregulated the expression of Drp1 after BLM treatment in MLE-12 cells, and these effects were reversed by transfection with si-Prdx3, indicating that YAP1 maintains the balance between mitochondrial fission and fusion by modulating Prdx3 expression (Fig. 6e). Accordingly, Prdx3 silencing in the background of YAP1 overexpression increased the production of cytoplasmic and mitochondrial ROS (Fig. 6f and Supplementary Fig. 2b). Additionally, the mitochondrial membrane potential (MMP) was dramatically reduced in Prdx3-depleted cells (Fig. 6g and Supplementary Fig. 2c). Moreover, Prdx3 silencing abrogated the effect of YAP1 on mitochondrial length and morphology (Fig. 6h). The Seahorse assays indicated that YAP1 restored the O_2_ consumption rate, whereas this effect was abolished by si-Prdx3 transfection (Fig. 6i). Taken together, these findings indicated that YAP1 inhibited cellular senescence and mitochondrial dysfunction by elevating the expression of Prdx3.

### Forced expression of Prdx3 suppresses pulmonary fibrosis in mice

**Fig. 7:**
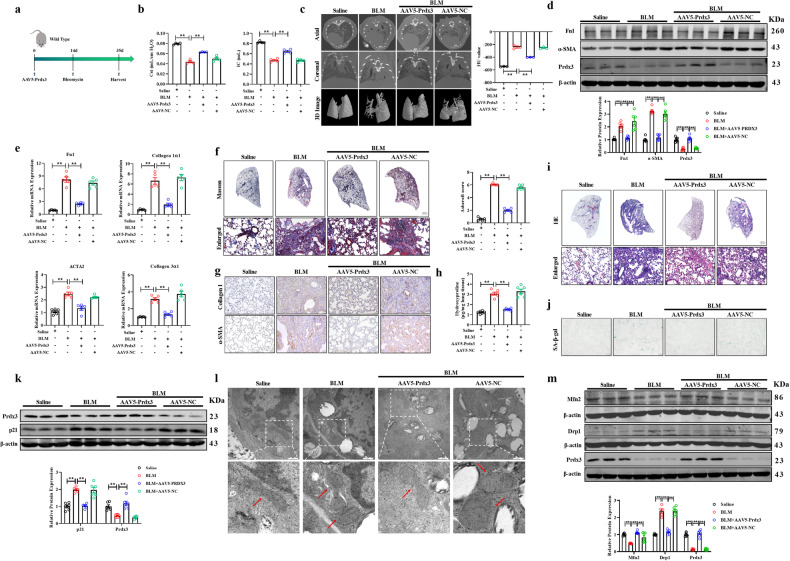
Prdx3 alleviates experimental pulmonary fibrosis in mice. **a** Schematic diagram of the procedure for overexpression of Prdx3 via AAV5 in the BLM-induced pulmonary fibrosis model. **b** Pulmonary function parameters, including Cst and IC, were measured in WT mice treated with AAV5-Prdx3 or AAV5-NC; *n* = 4. **c** Micro-CT images of the axial plane (upper panels) and the corresponding coronal plane (middle panels) in WT mice treated with AAV5-Prdx3 or AAV5-NC. The bottom panels show images of three-dimensional reconstructions of lung tissues based on the density; *n* = 3. **d** Western blot analysis of Fn1 and α-SMA protein expression in WT mice treated with AAV5-Prdx3 or AAV5-NC; *n* = 6. **e**The mRNA expression of Fn1, Collagen 1α1, Collagen 3α1, and ACTA2 in WT mice treated with AAV5-Prdx3 or AAV5-NC was measured by qRT–PCR; *n* = 5. **f**, **g** Images of Masson’s trichrome staining and immunohistochemical staining of paraffin-embedded lung sections from AAV5-Prdx3- or AAV5-NC-treated WT mice. Scale bars, 50 μm. **h** Hydroxyproline content in WT mice administered AAV5-Prdx3 or AAV5-NC; *n* = 6. **i** Images of HE staining in paraffin-embedded lung sections from AAV5-Prdx3- or AAV5-NC-treated WT mice. Scale bars, 50 μm. **j** SA-β-gal staining of frozen lung tissue slides. Scale bars, 50 μm. **k** Western blot analysis of p21 protein expression in WT mice treated with AAV5-Prdx3 or AAV5-NC; *n* = 6. **l** Transmission electron microscopy images of tissue samples from WT mice intratracheally administered AAV5-Prdx3 or AAV5-NC. Scale bars, 500 nm. **m** Western blot analysis of the protein expression of Drp1 and Mfn2 in WT mice administered AAV5-Prdx3 or AAV5-NC; *n* = 3–6. The data are presented as the means ± SEMs. **P < 0.01.

We next investigated whether the overexpression of Prdx3 prevents pulmonary fibrosis in mice. To this end, we treated WT mice with AAV5-Prdx3 14 days before BLM administration to increase Prdx3 expression in AT2 cells (Fig. 7a). Overexpression of Prdx3 restored the lung respiratory capacity and attenuated lung structural remodeling in BLM-treated mice (Fig. 7b, c). Moreover, forced expression of Prdx3 inhibited the BLM-induced upregulation of fibrosis-associated proteins and genes such as Fn1 and α-SMA (Fig. 7d, e). Furthermore, overexpression of Prdx3 decreased collagen deposition, α-SMA synthesis and the hydroxyproline content in the lungs of BLM-treated mice (Fig. 7f–h). In addition, HE staining demonstrated that AAV5-Prdx3 administration inhibited inflammatory infiltration in BLM-treated mice (Fig. 7i). Moreover, Prdx3 reduced the activity of senescence-associated β-gal and decreased the expression of p21 in mice after BLM induction (Fig. 7j, k). TEM revealed that Prdx3 overexpression ameliorated the loss of mitochondrial cristae loss and mitochondrial swelling (Fig. 7l). Moreover, overexpression of Prdx3 improved mitochondrial function by altering the expression levels of Drp1 and Mfn2 (Fig. 7m). In summary, Prdx3 inhibits pulmonary fibrosis by alleviating senescence and mitochondrial dysfunction in the lungs.

### Knockdown of Prdx3 facilitates pulmonary fibrosis in BLM-treated YAP1-cKI mice

**Fig. 8:**
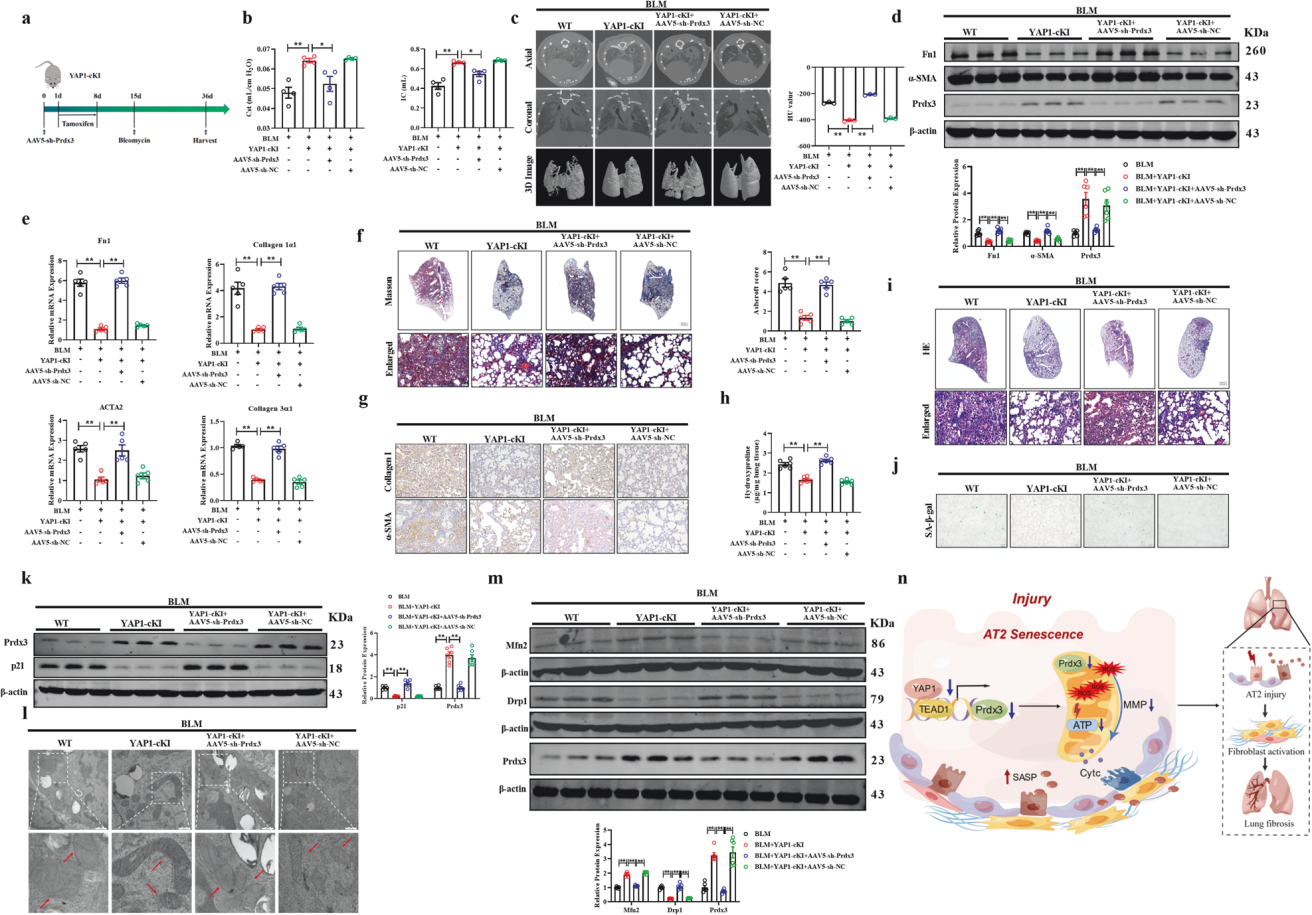
Silencing of Prdx3 promotes pulmonary fibrosis in BLM-treated YAP1-cKI mice. **a** Schematic of the experimental design showing the silencing of Prdx3 in YAP1-cKI mice treated with BLM. **b** Pulmonary function parameters, including Cst and IC, were measured in YAP1-cKI mice treated with AAV5-sh-Prdx3 or AAV5-sh-NC; *n* = 4. **c** Micro-CT images of the axial plane (upper panels) and corresponding coronal plane (middle panels) in YAP1-cKI mice treated with AAV5-sh-Prdx3 or AAV5-sh-NC. The bottom panels show images of three-dimensional reconstructions of lung tissues based on the density; *n* = 3. **d** Western blot analysis of Fn1 and α-SMA protein expression in YAP1-cKI mice treated with AAV5-sh-Prdx3 or AAV5-sh-NC; *n* = 6. **e** The mRNA expression of Fn1, Collagen 1α1, Collagen 3α1, and ACTA2 in YAP1-cKI mice treated with AAV5-sh-Prdx3 or AAV5-sh-NC was measured by qRT–PCR; *n* = 5. **f**, **g** Images of Masson’s trichrome staining and immunohistochemical staining in paraffin-embedded lung sections from YAP1-cKI mice treated with AAV5-sh-Prdx3 or AAV5-sh-NC. Scale bars, 50 μm. **h** The hydroxyproline content was measured in YAP1-cKI mice treated with AAV5-sh-Prdx3 or AAV5-sh-NC; *n* = 6. **i** Images of HE staining in lung paraffin sections of YAP1-cKI mice treated with AAV5-sh-Prdx3 or AAV5-sh-NC. Scale bars, 50 μm. **j** SA-β-gal staining of frozen lung tissue sections from AAV5-sh-Prdx3- or AAV5-sh-NC-treated YAP1-cKI mice. Scale bars, 50 μm. **k** Western blot analysis of p21 protein expression in YAP1-cKI mice treated with AAV5-sh-Prdx3 or AAV5-sh-NC; *n* = 6. **l** Transmission electron microscopy images of tissue samples from YAP1-cKI mice intratracheally injected with AAV5-sh-Prdx3 or AAV5-sh-NC. Scale bars, 500 nm. **m** Western blot analysis of the protein expression of Drp1 and Mfn2 in YAP1-cKI mice intratracheally injected with AAV5-sh-Prdx3 or AAV5-sh-NC; *n* = 6. **n** After exposure to injury stimuli, the expression of YAP1 in AT2 cells was significantly decreased, and YAP1 then lost its ability to bind to TEAD1 to upregulate Prdx3, an antioxidant enzyme in mitochondria. A decrease in Prdx3 expression led to ROS accumulation and mitochondrial dysfunction, which ultimately resulted in cellular senescence. Senescent AT2 cells secreted SASP factors to activate lung fibroblasts, which ultimately contributed to pulmonary fibrosis. This figure was generated by Figdraw. The data are presented as the means ± SEMs. *P < 0.05, **P < 0.01.

Next, we assessed the role of YAP1 and Prdx3 silencing in cellular senescence and mitochondrial dysfunction in vivo (Fig. 8a). Pulmonary function tests and micro-CT analysis demonstrated that Prdx3 silencing increased pulmonary fibrosis in BLM-treated YAP1-cKI mice, indicating that YAP1 inhibited BLM-induced pulmonary fibrosis through Prdx3 in vivo (Fig. 8b, c). Western blot and qRT‒PCR analyses revealed significantly upregulated expression of fibrosis-related proteins and genes such as Fn1 and α-SMA in Prdx3-depleted YAP1-cKI mice following BLM administration (Fig. 8d, e). Furthermore, Prdx3 knockdown resulted in collagen deposition and lung structural remodeling in BLM-treated YAP1-cKI mice (Fig. 8f–i). In addition, Prdx3 knockdown increased SA-β-gal activity in YAP1-cKI mice following BLM treatment, suggesting that Prdx3 silencing promoted cellular senescence in BLM-treated YAP1-cKI mice (Fig. 8j, k). As shown in Fig. 8l, the YAP1-cKI mice exhibited reductions in mitochondrial swelling and the loss of mitochondrial cristae induced by BLM, and this protective effect was abolished by the administration of AAV5-sh-Prdx3. The balance of mitochondrial fission and fusion was disrupted in BLM-treated YAP1-cKI mice after Prdx3 depletion (Fig. 8m). Taken together, these findings from our animal experiments suggested that overexpression of YAP1 in AT2 cells exerted antifibrotic effects by regulating Prdx3.

## Discussion

IPF is a chronic disease that is strongly correlated with age and has limited therapeutic options. Injury to the alveolar epithelium leads to the senescence and death of AT2 cells, resulting in the activation of an inflammatory response and repair program^20^. However, aberrant communication between endothelial cells, inflammatory cells and epithelial cells and fibroblasts promotes sustained fibroblast activation and ECM deposition^21^. In this study, we described the decreased expression of YAP1 in IPF patients and fibrotic mouse lungs. Our study showed that mice deficient in YAP1 in AT2 cells exhibited deterioration of lung respiratory function and exacerbation of pulmonary fibrosis following BLM administration. We further found that after BLM challenge, YAP1-cKI mice exhibited an improved pulmonary respiratory capacity and attenuation of pulmonary fibrosis. The effect of YAP1 on AT2 cells during pulmonary fibrosis depended on the modulation of cellular senescence and mitochondrial function mediated through targeting of Prdx3. We also identified the beneficial role of Prdx3 in vivo. Overexpression of Prdx3 suppressed experimental pulmonary fibrosis, whereas silencing Prdx3 partially abrogated the protective effect of YAP1. Hence, we elucidated that targeting the YAP1/TEAD1-Prdx3 axis may be an effective therapeutic strategy for pulmonary fibrosis (Fig. 8n).

YAP1 is a transcriptional coactivator regulated by the canonical Hippo signaling pathway. When Hippo signaling is unactivated, YAP1 translocates into the nucleus to bind to its partners, such as TEAD1, to activate downstream genes^22^. Previous studies have shown that YAP1 plays critical roles in tumorigenesis, inflammation, and organ fibrosis. Forced expression of the active form of YAP1 in mature mouse hearts was reported to promote cardiac repair and improve contractility following myocardial infarction^23^. In contrast, loss of YAP1/TAZ inhibits cardiac fibroblast activation to suppress unfavorable cardiac remodeling and improve cardiac function after myocardial infarction^24^. These results indicate that altered expression of YAP1 in different types of cells may lead to different disease outcomes. Regarding the effect of YAP1 on pulmonary fibrosis, research has shown that the inhibition of YAP/TAZ through dopamine receptor D1 in lung fibroblasts reduces pulmonary fibrosis^25^.

However, a previous study revealed aberrant activation of the Hippo/YAP signaling pathway in AT2 cells of IPF patients^26^, and Alexander et al. showed that increased expression of YAP in epithelial progenitor cells of the embryonic lung resulted in pulmonary lesions, a characteristic shared with IPF^27^; these findings were contradictory to our results. However, it has been reported that increased expression of YAP1 in AT2 cells after lung injury could promote lung regeneration^28^. Moreover, a study revealed that endothelial cell-derived S1P caused the nuclear translocation of YAP1 in AT2 cells and subsequently facilitated lung repair^29^. The reason for these contradictory findings may be because in different stages of IPF development, the Hippo/YAP pathway is activated to promote lung repair after insult. Another possible reason may be limitations inherent in the BLM-induced mouse model of pulmonary fibrosis: although the administration of a single dose of bleomycin directly into the lungs of mice is a commonly utilized experimental model for investigating pulmonary fibrogenesis, this model still exhibits significant differences from IPF in humans^30^. One limitation is that the predominant inflammatory phase occurs 3–7 days after BLM treatment and is characterized by the upregulation of acute inflammatory cytokines^31^, and spontaneous reversal of fibrosis then occurs 3–4 weeks post-intratracheal bleomycin administration^32^. Hence, further research is needed to elucidate the precise role of YAP1 in AT2 cells through which BLM-induced pulmonary fibrosis was inhibited in our study.

Cellular senescence is a consequence of replicative limitations or other precipitating factors, such as oxidative stress and mitochondrial dysfunction^33^. A previous study demonstrated a senescent phenotype of AT2 cells in IPF patients^2^. Senescent AT2 cells secrete proinflammatory cytokines through the activation of a program termed the SASP to activate lung fibroblasts. A recent report revealed that the suppression of AT2 cell senescence alleviated pulmonary fibrosis^34^, suggesting that inducing senescence in AT2 cells may be an effective therapeutic strategy for pulmonary fibrosis. YAP1 is a potent factor that inhibits senescence and promotes cell survival. It has been reported that YAP1 functions as a negative regulator of p21 to eliminate senescent cells^35^. In this study, we demonstrated decreased expression of p16 and p21 in the lungs of YAP1-cKI mice treated with BLM. Moreover, overexpression of YAP1 suppressed BLM-induced senescence in MLE-12 cells. This result was in accordance with a previous study showing that YAP1 restrained astrocyte senescence by targeting the expression of CDK6 in the context of Alzheimer’s disease^36^. However, researchers have shown that hyperactivation of YAP1 in primary human ovarian surface epithelial cells induces cellular senescence^37^, and this finding seems to contradict our results. Therefore, further research should be carried out to explore the complicated underlying mechanism involved.

Mitochondrial dysfunction is a common precipitating factor for most chronic diseases, including pulmonary fibrosis^38^. Moreover, mitochondrial dysfunction is a main cause of senescence, and mitochondrial aberrations, such as the loss of cristae and destruction of the inner membrane, are often observed in senescent cells^39^. Increasing evidence has revealed that damage to mitochondria may cause senescent cell cycle arrest, and mitochondrial dysfunction can lead to premature senescence in T cells^40^. Hence, restoring mitochondrial function may inhibit cellular senescence.

Peroxiredoxins are the most abundant superfamily of peroxidases^41^. Prdx3, an important peroxiredoxin located mainly in mitochondria, can eliminate superoxide radical anions and hydrogen peroxide, which are generally collectively referred to as ROS^42^. It is well known that mitochondria are the dominant source of ROS^43^—in the physiological state, ROS function as important cellular signaling molecules; however, excessive accumulation of ROS disrupts the redox balance and contributes to mitochondrial dysfunction and DNA damage^44^. A recent report demonstrated the protective effect of Prdx3 on mitochondrial dysfunction during cerebellar ataxia^45^. Furthermore, Prdx3 is a crucial downstream target of APN/SIRT3 signaling that inhibits mitochondrial dysfunction after traumatic brain injury^46^. Through in vitro and in vivo experiments, we found that Prdx3 maintained the balance between mitochondrial fission and fusion, restored the mitochondrial membrane potential, and reduced ROS production, which eventually suppressed BLM-induced senescence in AT2 cells. Silencing of Prdx3 abolished the beneficial effects of YAP1, indicating the vital role of the YAP1/TEAD1-Prdx3 axis in inhibiting cellular senescence and maintaining cell survival, whereas overexpression of Prdx3 in AT2 cells alleviated pulmonary fibrosis. Overall, our study revealed that the YAP1/TEAD1-Prdx3 axis inhibits lung senescence, promotes cell survival and lung repair, and alleviates pulmonary fibrosis.

In this study, we first revealed that increased expression of YAP1 in AT2 cells mitigated BLM-induced pulmonary fibrosis and revealed the regulatory relationship between YAP1/TEAD1 and Prdx3. Moreover, our study revealed that the YAP1/TEAD1-Prdx3 axis acts as a novel antifibrotic mediator by inhibiting the senescence of AT2 cells and maintaining alveolar epithelial homeostasis, consistent with recent studies showing that YAP1 is critical for AT2 cell regeneration during lung inflammation and injury^47^. However, approaches for elevating the expression of YAP1 are limited because of the lack of agonists of YAP1. Hence, AAV-mediated gene therapy may become a novel strategy for delivering YAP1. Recent studies have demonstrated the application of AAV-DTR/DT to establish an acute lung injury model, and an AAV carrying proSFTPB cDNA was shown to significantly restore surfactant homeostasis, inhibit lung injury and improve pulmonary function in SFTPB-deficient mice^48^. In this study, we utilized AAV5 to increase the expression of Prdx3, but the curative effect of AAV-mediated YAP1 transfer needs further validation in clinical trials.

Overall, our research demonstrated that YAP1 attenuated the chronic injury-induced senescence of AT2 cells and inhibited pulmonary fibrosis. This effect was exerted by alleviating mitochondrial dysfunction and oxidative stress through targeting of Prdx3.

### Supplementary information

Supplementary materials
