# Supplementary material for: YAP1 inhibits the senescence of alveolar epithelial cells by targeting Prdx3 to alleviate pulmonary fibrosis
Source: Exp Mol Med. 2024 Jul 1;56(7):1643–54. doi: 10.1038/s12276-024-01277-0 (PMC11297023; doi:10.1038/s12276-024-01277-0)
Supplement: Supplementary file 1 — Supplementary materials [file 12276_2024_1277_MOESM1_ESM.pdf]

Supplementary Materials

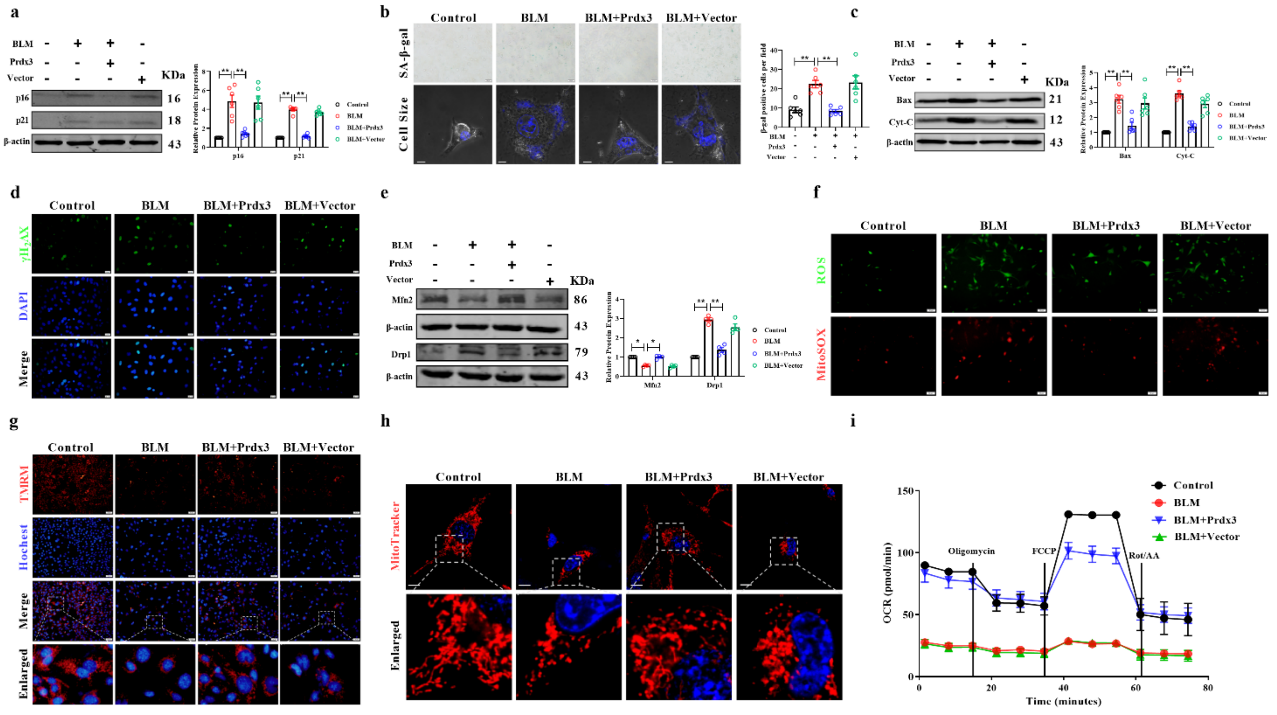

Supplementary Fig. 1 Overexpression of Prdx3 inhibits BLM-induced senescence and mitochondrial dysfunction.

(a) Western blot results of protein expression of p16 and p21 in MLE-12 cells transfected with Prdx3 after BLM treatment, *n*=6. (b) SA-β-gal staining for cellular senescence of MLE-12 cells transfected with Prdx3 after BLM treatment, *n*=6. Scale bars, 50 μm. The cell size was determined by confocal microscopy; nuclei were stained with Hochest 33342 (blue). Scale bars: 10 μm. (c) Western blot results of protein expression of Bax and Cyt-C in MLE-12 cells transfected with Prdx3 after BLM treatment, *n*=6. (d) Immunofluoresence staining assay of γH_2AX in MLE-12 cells treated with Prdx3 plasmid. Scale bars: 20 μm. (e) The protein level of Drp1, and Mfn2 was determined by western blot, *n*=4. (f) Cytosolic and mitochondrial ROS in Prdx3 over-expressing MLE-12 cells after BLM administration was assessed by DCFH-DA probe and MitoSOX. Scale bars: 50 μm. (g) Representative images obtained from MLE-12 cells stained with TMRM (red) and Hoechst 33342 for nuclei (blue). Scale bars: 50 μm. (h) Representative images acquired by confocal microscopy showing the morphological change of mitochondria. Scale bars: 10 μm. (i) O_2 consumption rate was assessed using a seahorse mitochondrial stress test assay. Data are presented as the mean ± SEM. $^{**}P < 0.01$ (Ordinary one-way ANOVA).

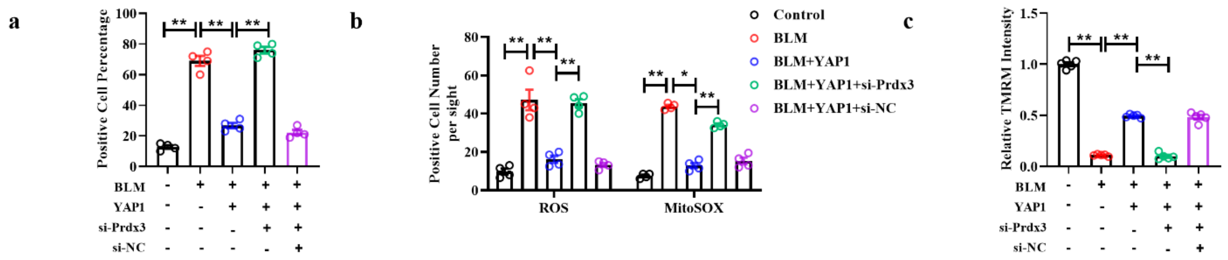

Supplementary Fig. 2 Silencing Prdx3 restores cellular senescence and mitochondrial dysfunction inhibited by YAP1.

(a) Statistics of γH_2AX in MLE-12 cells treated with YAP1 or silencing Prdx3 following BLM induction. *n*=4. (b) Statistics of ROS and MitoSOX in Prdx3 over-expressing MLE-12 cells after BLM administration. *n*=4. (c) Statistics of MLE-12 cells stained with TMRM. *n*=5. $^{*}P < 0.05$, $^{**}P < 0.01$.
